# Prognostic nomograms for predicting overall survival and cancer-specific survival of patients with very early-onset colorectal cancer: A population-based analysis

**DOI:** 10.17305/bjbms.2021.7035

**Published:** 2022-03-27

**Authors:** Bingtian Dong, Yuping Chen, Guorong Lyu

**Affiliations:** 1 Department of Ultrasound, The Second Affiliated Hospital of Fujian Medical University, Quanzhou, Fujian, China; 2 Department of Endocrinology, The Second Affiliated Hospital of Fujian Medical University, Quanzhou, Fujian, China; 3 Department of Clinical Medicine, Quanzhou Medical College, Quanzhou, Fujian, China

**Keywords:** Very early-onset colorectal cancer, prognostic nomogram, overall survival, cancer-specific survival, SEER

## Abstract

In contrast to the declining incidence in older populations, the incidence of very early-onset colorectal cancer (VEO-CRC) patients (aged ≤40 years) has been increasing in different regions of the world. In this study, we aimed to establish nomogram models for the prognostic prediction of patients with VEO-CRC for both overall survival (OS) and cancer-specific survival (CSS). Patients diagnosed with VEO-CRC between 2010 and 2015 from the Surveillance, Epidemiology, and End Results (SEER) database were collected and randomly assigned to the training cohort and validation cohort at a ratio of 7:3 for model construction and internal validation. Using univariate and multivariate Cox regression analysis to screen important variables, which were then used to construct a nomogram. The nomogram was evaluated using calibration curves and the receiver operating characteristic (ROC) curves. A total of 3061 patients were included and randomly divided into the training cohort (n = 2145) and validation cohort (n = 916). Five independent prognostic factors, including race, grade, tumor size, American Joint Commission on Cancer (AJCC) stage, and AJCC T stage, were all significantly identified in OS multivariate Cox regression analysis. Meanwhile, in CSS, multivariate Cox regression analysis demonstrated that race, grade, tumor size, AJCC stage, AJCC T stage, AJCC N stage, and SEER stage were independent prognostic factors. The calibration plots of the established nomograms indicated high correlations between the predicted and observed results. C-index and ROC analysis implied that our nomogram model has a strong predictive ability. Moreover, nomograms also showed higher C-index values compared to tumor-node-metastasis and SEER stages. We established and validated a simple-to-use nomogram to evaluate the 1-, 3-, and 5-year OS and CSS prognosis of patients with VEO-CRC. This tool can assist clinicians to optimize individualized treatment plans.

## INTRODUCTION

Colorectal cancer (CRC) is the third most common cancer (following lung and breast cancer) and one of the leading causes of cancer-related death worldwide in both genders [1,2]. It has been estimated that in 2020, approximately 1.9 million new cases of CRC were diagnosed and a total of 935,000 deaths from CRC occurred worldwide [3].

Decreasing incidence rates of CRC have been observed in persons aged 50 years and above [4]. However, an opposite trend appears among younger adults [5]. It is noteworthy that the largest increase in CRC incidence occurred among subjects aged 40 years or younger, which was defined as very early-onset CRC (VEO-CRC) [4,6]. Hence, there is an urgent need to identify crucial prognostic factors specifically for VEO-CRC patients, thus contributing to improved prediction of survival outcome as well as further clinical decision-making.

Clinically, the tumor lymph node metastasis (TNM) staging system, proposed by the American Joint Commission on Cancer (AJCC), is widely used to predict the prognosis of various cancers, but has some shortcomings and is deficient in predicting prognosis accurately [7]. In recent years, some studies have suggested that other factors, including primary site, tumor size, and marital status, may also influence the outcome of CRC patients [8,9]. Therefore, it is necessary to conduct a more comprehensive prognostic analysis for VEO-CRC patients based on all the risk factors related to cancer.

In the past decade, nomograms have acquired a wide acceptance as a unique, reliable method for predicting tumor prognosis in an individualized manner [10]. Based on a multivariable linear regression model, nomograms integrate multiple clinical predictors and show quantitative relationships between these individual predictors, which can accurately predict the overall survival (OS) and cancer-specific survival (CSS) for a single patient, assisting clinicians to optimize individualized therapeutic options and assess treatment outcomes [11]. Such studies have recently been reported in various types of cancers [7,11-13], but the research focusing on the OS and CSS prognosis of VEO-CRC is rarely reported.

The Surveillance, Epidemiology, and End Results (SEER) database, supported by the National Cancer Institute, collects data from 18 cancer registries and covers about one-third of the population in the US [14]. Basing studies on a wide-reaching, multicenter database can offer more compelling evidence compared to single-center studies [15]. In this study, a set of patients with VEO-CRC from the SEER database were chosen to recognize significant factors and a simple-to-use nomogram was established for predicting the 1-, 3-, and 5-year OS and CSS prognosis of VEO-CRC patients.

## MATERIALS AND METHODS

### Patient cohorts

Data were extracted from the SEER database using SEER*Stat software version 8.3.9. In this study, we selected young adult patients with VEO-CRC in the SEER database registered from 2010 to 2015, which includes clinicopathological and individualized prognosis data. Patients with VEO-CRC were identified by the International Classification of Diseases for Oncology, Third Edition site code (C18.0, C18.2-C18.7, C19.9, and C20.9) and the cancer staging scheme (version 0204). Inclusion criteria were as follows: (1) Patients aged ≤40 years old with a diagnosis of CRC; (2) complete survival information; (3) with surgery performed; (4) CRC was the only primary cancer; (5) without unknown race, grade, tumor size, and tumor stage; and (6) without missing information in SEER cause-specific death classification. In this study, we used the “caret” package in R programming language to randomly divide patients with VEO-CRC from the SEER database into the training cohort and validation cohort at a ratio of 7:3, for the construction and verification of the nomogram, respectively.

### Clinical variables

The following clinical variables were extracted from the SEER database: Age, sex, race, marital status, primary site, grade, histology, liver metastasis, lung metastasis, bone metastasis, brain metastasis, tumor size, TNM stage, and SEER stage. The TNM stage was determined according to the AJCC guidelines, 7^th^ edition. OS was defined as the date of diagnosis to the end of follow-up or death from all causes. In addition, the CSS analyzed in our study was defined as the survival time from diagnosis to death from CRC, excluding other causes. The primary endpoint of the clinical outcome was OS and the secondary endpoint was CSS.

### Ethical statement

The authors obtained authorization to access the SEER Research Data supported by the National Cancer Institute with reference number 19776-November 2020. Because public and anonymous data from the SEER database were used, informed patient consent was not required.

### Nomogram development and statistical analysis

Basic characteristics were presented as number (n) and percentage (%). X-tile software version 3.6.1 was used to assess the appropriate cutoff values for age and tumor size variables [16]. The Kaplan–Meier method and log-rank test were used to analyze the OS and CSS of VEO-CRC patients. Chi-square test was used for the comparison of categorical variables between the training cohort and validation cohort. Univariate and multivariate Cox regression analyses were utilized to identify variables that significantly associated with OS and CSS of VEO-CRC in the training cohort. In the Cox regression model, enter method was used. Hazard ratios (HRs) with 95% confidence intervals (CIs) for the effect of each predictor on OS and CSS were calculated.

The R language “rms” package was then used to construct the prognostic nomogram based on risk factors which were statistically significant in the multivariate analysis to predict the 1-, 3-, and 5-year OS and CSS prognosis of VEO-CRC patients. Using the area under the receiver operating characteristic (ROC) curve (AUC) computed with the R language “pROC” package, we evaluated the discrimination ability of nomograms [17]. Moreover, concordance index (C-index) and calibration curve analysis were used to evaluate the accuracy and reliability of the nomogram, and compared with that of TNM stage and SEER stage [18,19]. To evaluate the calibration and discrimination of the nomogram, a validation cohort was then devoted to validate the prognostic nomogram. Finally, we measured the applicability of the nomogram to clinical practice through decision curve analysis (DCA) using the R language “rmda” package [20]. All statistical analyses were performed with R statistical software (version 4.0.4, R Foundation for Statistical Computing, Vienna, Austria). *p* < 0.05 was considered statistically significant.

## RESULTS

### Demographic and pathologic characteristics

**FIGURE 1:**
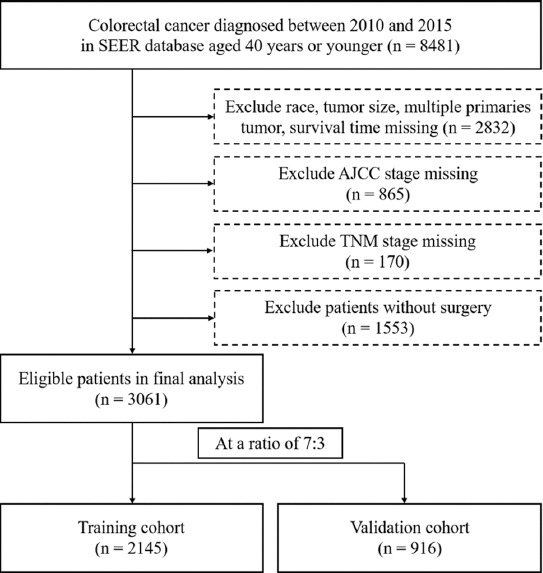
Flow diagram of patient selection criteria. According to the criteria, 3061 patients were collected from the SEER database and randomly assigned into the training cohort (n = 2145) and validation cohort (n = 916) at a ratio of 7:3.

Following inclusion criteria, a total of 3061 eligible patients diagnosed with VEO-CRC between 2010 and 2015 from the SEER database were collected in this study, with 2145 assigned to the training cohort and 916 to the validation cohort randomly, for the construction and verification of the nomogram, respectively (Figure 1). Demographic and clinical characteristics of VEO-CRC patients are listed in Table 1.

**TABLE 1:**
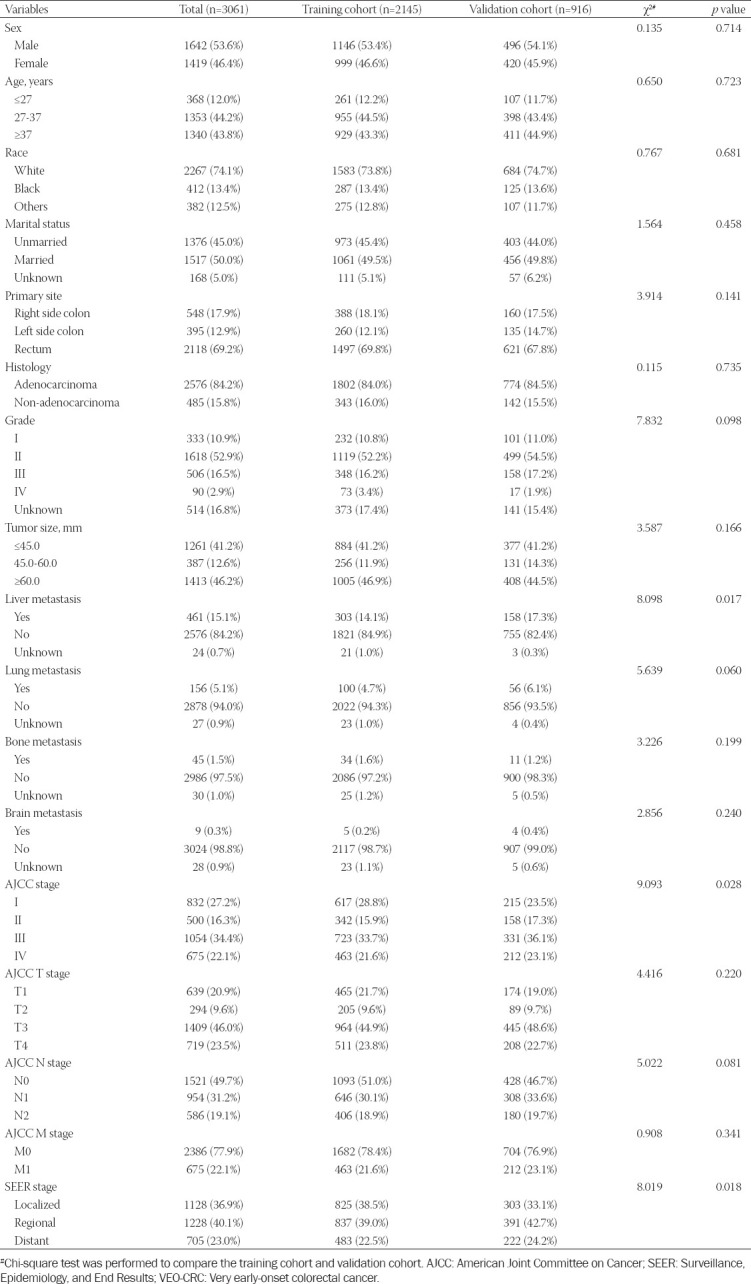
Baseline demographic and clinical characteristics with VEO-CRC patients

Among all patients, 53.6% were male and 46.4% female; 50.0% were married and 45.0% unmarried. The majority of race was White, accounting for 74.1% of all the patients. The rectum (69.2%) was the most common primary tumor site, followed by the right side colon (17.9%) and left side colon (12.9%). In addition, the majority of the cohort had the adenocarcinoma histological type (84.2%). In total, 15.1% of the patients had liver metastasis, 5.1% with lung metastasis, 1.5% with bone metastasis, and 0.3% with brain metastasis. For AJCC TNM stage, Stage III was the most common type (34.4%), followed by Stage I (27.2%), Stage IV (22.1%), and Stage II (16.3%). About 40.1% of all the patients were regional in SEER stage, 36.9% were localized, and 23.0% were distant.

**FIGURE 2:**
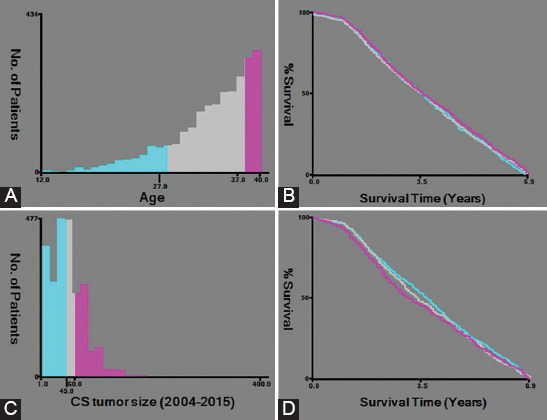
The X-tile analysis of appropriate cutoff values for age and tumor size variables. (A, B) The appropriate cutoff values of age were 27 and 37 years. (C, D) The appropriate cutoff values of tumor size were 45.0 and 60.0 mm.

The appropriate cutoff values for age and tumor size variables were determined by X-tile software (Figure 2). Specifically, for all the VEO-CRC patients, 1353 were between 27 and 37 years old (44.2%) and 1340 were ≥37 years old (43.8%), whereas 368 were aged 27 years old or younger (12.0%). For tumor size, 60.0 mm was the most common type (46.2%) followed by 45.0 mm (41.2%) and 45.0-60.0 mm (12.6%).

### Identification of independent prognostic factors of OS and CSS in training cohort

Using univariate Cox regression analysis in the training cohort, the results indicated that race, marital status, histology, grade, tumor size, liver metastasis, lung metastasis, bone metastasis, brain metastasis, AJCC stage, AJCC T stage, AJCC N stage, AJCC M stage, and SEER stage were significantly associated with OS (Table 2). Next, five independent prognostic factors, including race, grade, tumor size, AJCC stage, and AJCC T stage, were all significantly identified in OS multivariate Cox regression analysis (Table 2).

**TABLE 2:**
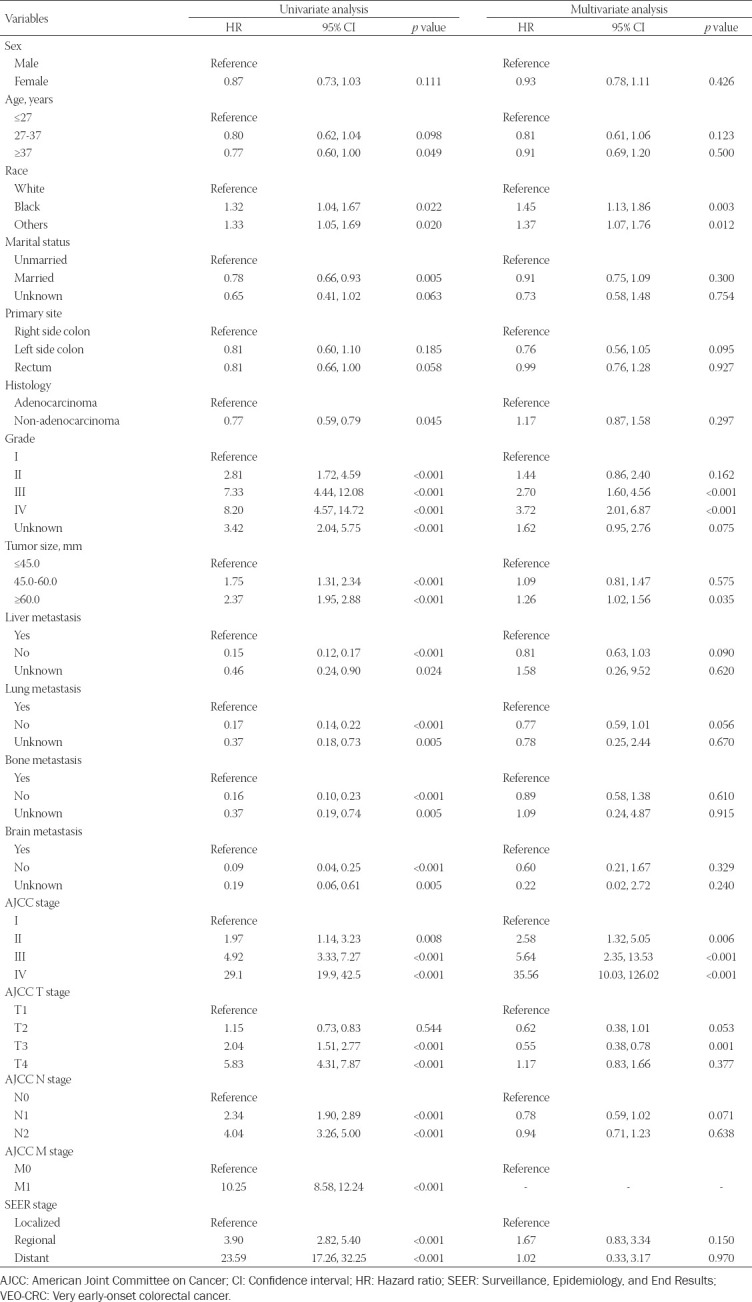
Univariate and multivariate analysis of overall survival in the training cohort

Meanwhile, in CSS, results of the univariate Cox regression analysis in the training cohort showed that race, marital status, primary site, grade, tumor size, liver metastasis, lung metastasis, bone metastasis, brain metastasis, AJCC stage, AJCC T stage, AJCC N stage, AJCC M stage, and SEER stage were significantly associated with CSS. Further multivariate Cox regression analysis demonstrated that race, grade, tumor size, AJCC stage, AJCC T stage, AJCC N stage, and SEER stage were independent prognostic factors associated with CSS (Table 3).

**TABLE 3:**
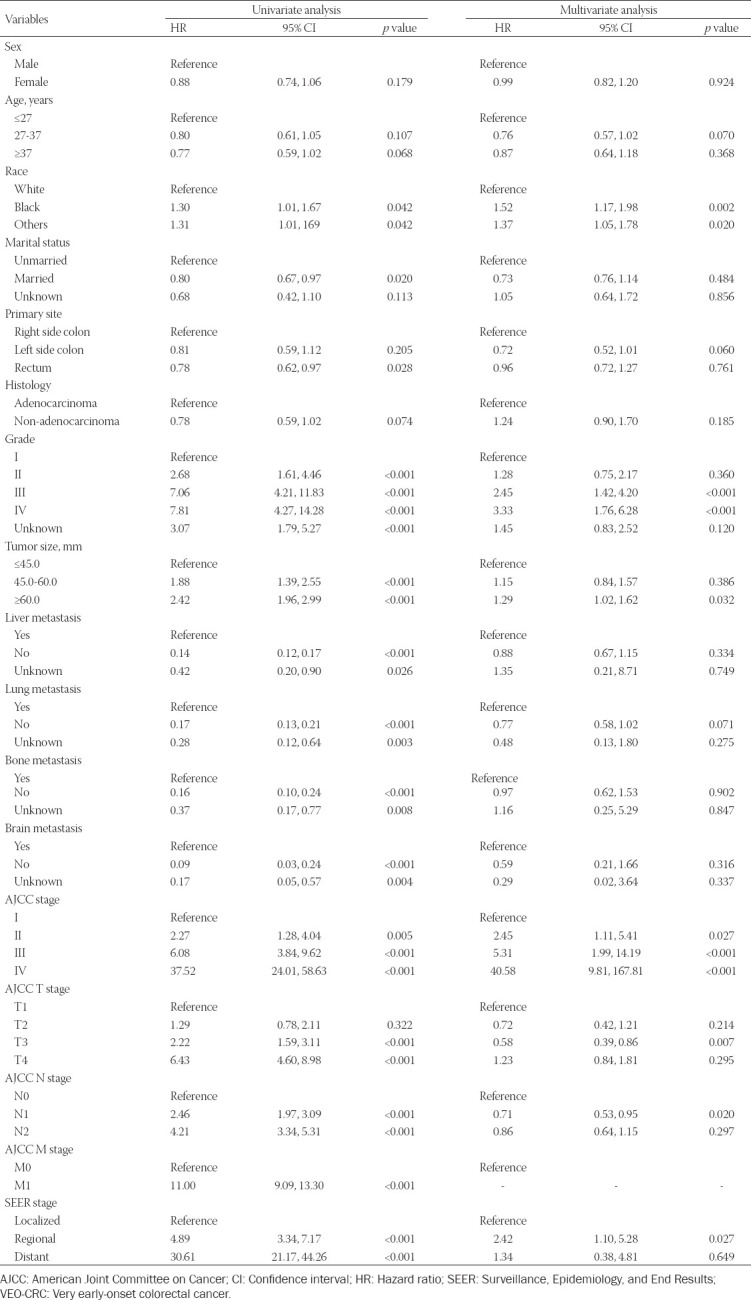
Univariate and multivariate analysis of cancer-specific survival in the training cohort

### Construction of the prognostic nomogram

**FIGURE 3:**
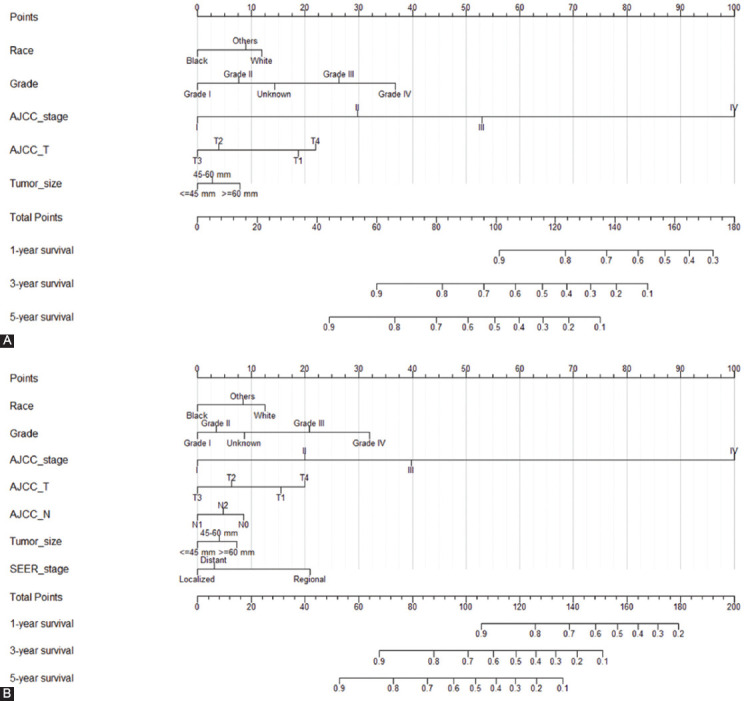
Establishment of overall survival (OS) and cancer-specific survival (CSS) nomograms. (A) Construction of OS nomogram; (B) construction of CSS nomogram.

Based on clinical variables which were statistically significant in the multivariate Cox regression results, the nomograms to predict the 1-, 3-, and 5-year OS and CSS prognosis of VEO-CRC patients were established (Figure 3). The nomogram for predicting the 1-, 3-, and 5-year OS prognosis of VEO-CRC patients contained the following independent prognostic factors: Race, grade, tumor size, AJCC stage, and AJCC T stage. According to HR, each variable corresponds to a score, which can be obtained by projecting to the top “points” axis. As a result, the total points are then obtained by summing the corresponding scores for each variable. Finally, the 1-, 3-, and 5-year OS can be estimated by projecting the total points to the bottom “1-year survival,” “3-year survival,” and “5-year survival” axis. Moreover, a prognostic nomogram for predicting the 1-, 3-, and 5-year CSS prognosis of VEO-CRC patients was established as well, which consisted of race, grade, tumor size, AJCC stage, AJCC T stage, AJCC N stage, and SEER stage as the prognostic factors.

For example, using the OS nomogram, a White patient (12 points) with T4 stage (22 points), AJCC TNM Stage III (53 points), Grade III (27 points), and tumor size >6 cm (8 points) would have a total of 122 points, which means a predicted 1-year OS of approximately 80%, predicted 3-year OS of approximately 40%, and predicted 5-year OS of approximately 20%.

### Validation and calibration of the prognostic nomogram

**FIGURE 4:**
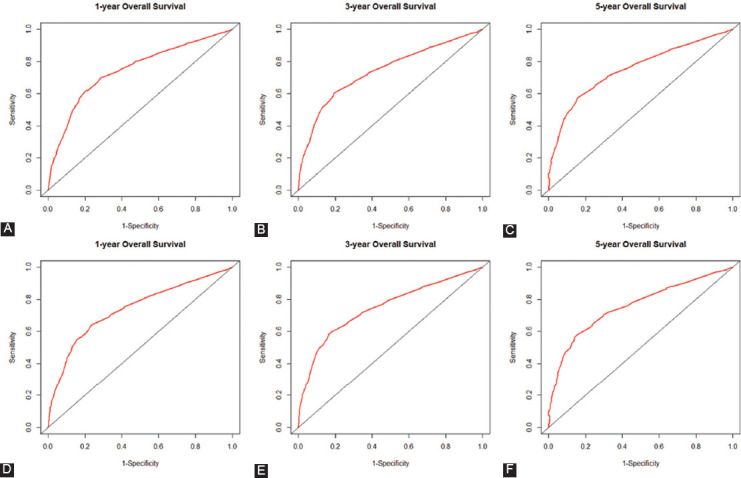
Receiver operating characteristics curve (ROC) comparison of overall survival (OS) nomogram. (A) One-year ROC of OS nomogram using training cohort; (B) 3-year ROC of OS nomogram using training cohort; (C) 5-year ROC of OS nomogram using training cohort; (D) 1-year ROC of OS nomogram using validation cohort; (E) 3-year ROC of OS nomogram using validation cohort; (F) 5-year ROC of OS nomogram using validation cohort.

**FIGURE 5:**
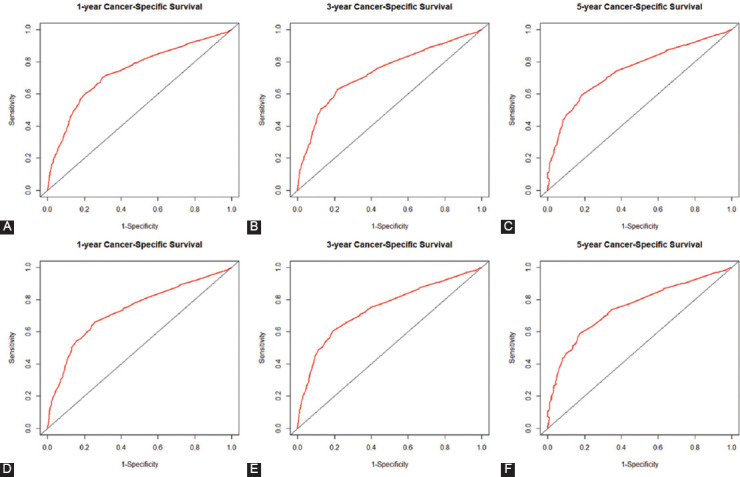
Receiver operating characteristics curve (ROC) comparison of cancer-specific survival (CSS) nomogram. (A) One-year ROC of CSS nomogram using training cohort; (B) 3-year ROC of CSS nomogram using training cohort; (C) 5-year ROC of CSS nomogram using training cohort; (D) 1-year ROC of CSS nomogram using validation cohort; (E) 3-year ROC of CSS nomogram using validation cohort; (F) 5-year ROC of CSS nomogram using validation cohort.

**SUPPLEMENTARY FIGURE S1:**
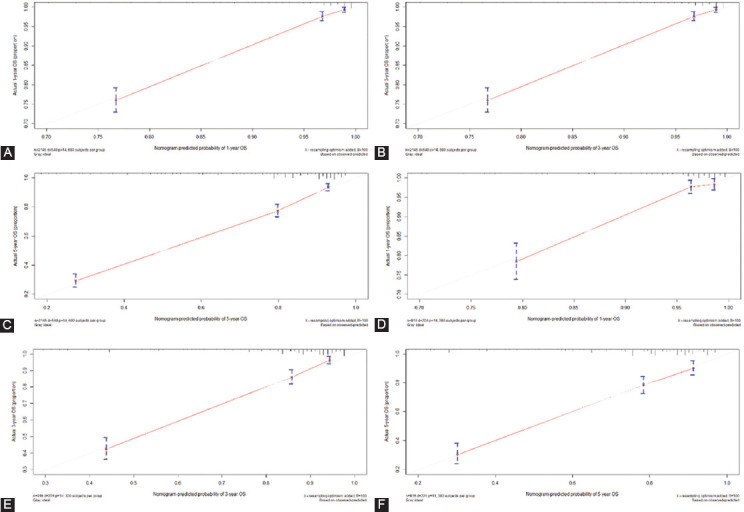
Calibration plots of overall survival (OS) nomogram model. (A) One-year calibration plot of OS using training cohort; (B) 3-year calibration plot of OS using training cohort; (C) 5-year calibration plot of OS using training cohort; (D) 1-year calibration plot of OS using validation cohort; (E) 3-year calibration plot of OS using validation cohort; (F) 5-year calibration plot of OS using validation cohort.

**SUPPLEMENTARY FIGURE S2:**
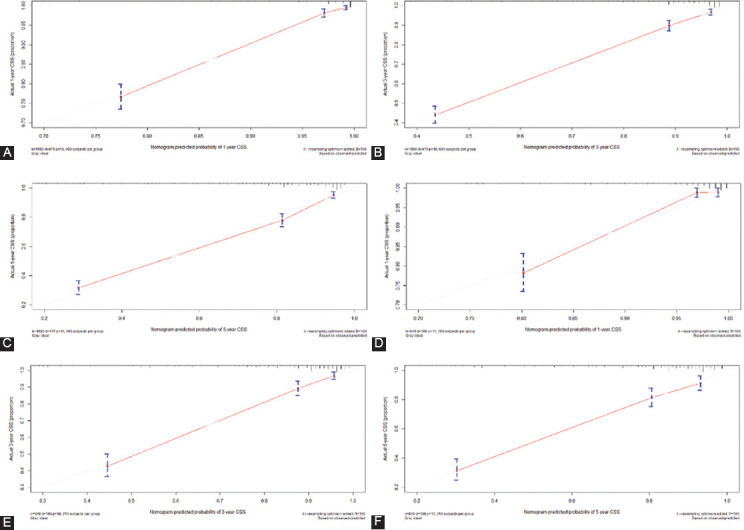
Calibration plots of cancer-specific survival (CSS) nomogram model. (A) One-year calibration plot of CSS using training cohort; (B) 3-year calibration plot of CSS using training cohort; (C) 5-year calibration plot of CSS using training cohort; (D) 1-year calibration plot of CSS using validation cohort; (E) 3-year calibration plot of CSS using validation cohort; (F) 5-year calibration plot of CSS using validation cohort.

Using the C-index value and AUC value, we evaluated the discrimination ability of established OS and CSS nomograms. Specifically, C-index of the OS nomogram was 0.842 (95% CI: 0.826-0.858) in training cohort while 0.819 (95% CI: 0.792-0.846) in the validation cohort (Table 4). The C-index of the CSS nomogram was 0.853 (95% CI: 0.835-0.871) and 0.838 (95% CI: 0.813-0.863) in the training cohort and validation cohort, respectively. Calibration plots were used to assess the calibration of our nomograms. The calibration plots of the established 1-, 3-, and 5-year OS and CSS nomograms in the training cohort and validation cohort indicated high correlations between the predicted and observed results (Supplementary Figures S1 and S2). In the ROC curve analysis, the 1-, 3-, and 5-year AUC values of the OS nomogram were 0.745, 0.740, and 0.751, respectively, in the training cohort, corresponding to 0.739, 0.747, and 0.756 in the validation cohort (Figure 4). Meanwhile, in CSS, the 1-, 3-, and 5-year AUC values of the nomogram were 0.739, 0.737, and 0.748, respectively, in the training cohort, corresponding to 0.735, 0.744, and 0.752 in the validation cohort (Figure 5). In this study, we found that both the 1-, 3-, and 5-year AUC values of the OS and CSS nomograms were around 0.75, indicating that the constructed nomograms have good discriminatory ability for OS and CSS prediction.

**TABLE 4:**
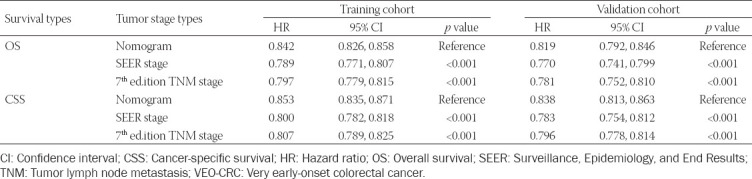
Comparison of C-indexes between the nomograms, TNM, and SEER stages in patients with VEO-CRC

### Clinical utility

**FIGURE 6:**
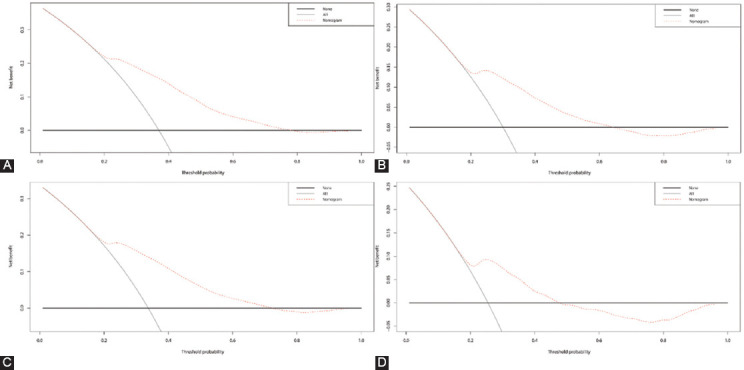
Decision curve analysis (DCA) curves of the nomograms for overall survival (OS) and cancer-specific survival (CSS) in both training and validation cohorts. (A) The DCA curve of nomogram for OS in training cohort; (B) the DCA curve of nomogram for CSS in training cohort; (C) the DCA curve of nomogram for OS in validation cohort; (D) the DCA curve of nomogram for CSS in validation cohort. X-axis and Y-axis represent threshold probability and net benefit, respectively. The most favorable threshold probabilities for predicting OS and CSS in the training cohort with the nomogram were 0.2-0.7 and 0. 2-0.6, respectively.

DCA is an advanced method that is used to analyze the net clinical benefits of predictive models. In this study, we evaluated the clinical applicability of established OS and CSS nomograms through DCA (Figure 6). The results showed that the most favorable threshold probabilities for predicting OS and CSS in the training cohort with the nomogram were 0.2-0.7 and 0.2-0.6, respectively. As demonstrated by the favorable threshold probability, it indicated that the nomogram had a satisfactory clinical benefit for the management of VEO-CRC and can assist clinicians to predict OS and CSS accurately.

### Comparison of the nomograms with the AJCC TNM stage and SEER stage

Using the C-index, we compared the nomograms, the AJCC TNM stage, and the SEER stage. In both training cohort and validation cohort, the OS and CSS nomograms constructed in this study exhibited better results to AJCC TNM stage and SEER stage (Table 4).

## DISCUSSION

This study established and validated a simple-to-use nomogram to evaluate the 1-, 3-, and 5-year OS and CSS prognosis of patients with VEO-CRC based on the public database SEER. Compared with TNM stage and SEER stage, the nomogram exhibited a better predictive performance and can be used to assist clinicians to optimize individualized treatment plans for VEO-CRC patients.

This is particularly alarming as incidence of CRC in young adults has been increasing. Compared to older patients, patients with VEO-CRC (aged ≤40 years) suffer from more aggressive tumor biology and are at more advanced disease stages when they were diagnosed [5]. Thus, it is clinically meaningful to establish a robust predictive model to accurately predict the survival time of VEO-CRC patients by comprehensively considering multiple prognostic factors. To the best of our knowledge, nomogram models for VEO-CRC that incorporated demographic and clinicopathological variables are not available to date. Based on a substantial population size from the SEER database, we used significant independent prognostic factors to establish and validate a simple-to-use nomogram for predicting the 1-, 3-, and 5-year OS and CSS prognosis of individual VEO-CRC patients.

Interestingly, in OS, five independent prognostic factors (race, grade, tumor size, AJCC stage, and AJCC T stage) out of 17 variables were determined for the construction of nomogram to predict the 1-, 3-, and 5-year prognosis of VEO-CRC patients. Meanwhile, in CSS, a prognostic nomogram was established as well, which consisted of race, grade, tumor size, AJCC stage, AJCC T stage, AJCC N stage, and SEER stage as the prognostic factors. These variables which were associated with the prognosis of CRC have been reported in the previous studies. Our present study showed that race was an independent prognostic variable affecting OS and CSS in patients with VEO-CRC, which was in agreement with previous reports [21]. In fact, race has been viewed as one of the risk factors for the prognosis of various cancers [22]. It is widely recognized that genetic differences among different races are also a significant risk factor for tumor prognosis [23]. We found that the grade of the tumor also significantly affects the OS and CSS prognosis of patients with VEO-CRC. An increase in tumor pathological grade indicates that the malignancy of the tumor is increasing [24]. In addition, the tumor pathological grade was positively correlated with the tumor invasiveness [25]. It has been proposed that in high-grade tumors, cancer cells were insensitive to treatment [26], thus adversely affecting the prognosis of patients.

In our study, tumor size was an independent prognostic variable both in the OS and CSS nomograms. Compared with tumor <4.5 cm, only the tumor >6.0 cm displayed significant higher prognostic risk, whereas the rest stratification remained insignificant. Tumor size could potentially be served as one of the insightful variables for the prediction of OS and CSS prognosis of patients with VEO-CRC. A number of studies have demonstrated tumor size as a negative variable for the prognostic risk prediction. In the study by Dai et al. [27], tumor size was proved to be a critical clinical factor in T1 colon cancer with considerable predictive value, outperformed any other prognostic clinical features in CSS prediction. Moreover, Saha et al. [28] suggested a significant positive correlation between tumor size and tumor pathological grade, and with T stage, whereas a negative correlation was found between tumor size and survival. Our findings were consistent with these previous reports. It is worth mentioning that in this study, X-tile tool was used to determine the appropriate cutoff values for age and tumor size variables. By constructing a two-dimensional projection, X-tile tool can illustrate potential subsets (cutoff) [16]. As a powerful graphic method, this tool has been widely used in many previous investigations [7,12,13,29]. To date, the role of tumor size in prognosis prediction of CRC has been intensively investigated [30]. Nevertheless, the appropriate cutoff value for tumor size variable in CRC remains largely arbitrary [31]. Hence, the introduction of X-tile for tumor size classification has several distinct advantages, including reliability and replicability [31].

Notably, sex, primary tumor site, and marital status were not an independent prognostic variable for patients with VEO-CRC in this study. In fact, sex has been considered as one of the essential clinical variables for cancer treatment [22]. In patients with CRC, survival outcome is different for different primary tumor location [21]. So far, some studies have intensively studied the tumor site of CRC in a large population [32,33]. In Petrelli’s meta-analysis [34], authors got a conclusion that left-sided colon cancers had a statistically significant better OS rate than right-sided colon cancers. However, the exact reason for this remains unknown yet. Moreover, the specific primary site of the tumor was not investigated in previous studies. Based on the SEER program, several studies examined the role of marital status in cancer [35]. There are several features for married cancer patients, including less metastatic diseases, more likely to receive definitive therapy, and reduced cancer-specific deaths [35]. As for colon cancer, married patients were associated with a significantly lower risk in predicting CSS prognosis [36]. Interestingly, although marriage was significantly associated with OS and CSS prognosis in univariate Cox regression analysis in the present study, it remained insignificant in multivariate Cox regression analysis.

Furthermore, we conducted a comprehensive evaluation of the performance of the OS and CSS nomograms established in this study. Both the OS and CSS nomograms showed good discriminatory ability with C-index values of 0.842 and 0.853, respectively. In the ROC curve analysis, both the 1-, 3-, and 5-year AUC values of the OS and CSS nomograms were around 0.75. In addition, the calibration plots of the established nomograms displayed barely any deviations from the reference line, which illustrates high correlations between the predicted and observed results. Moreover, in the validation cohort, the same results were also observed. These results implied that our nomogram model has a strong predictive ability. Compared to AJCC TNM stage and SEER stage, the nomogram exhibited better discrimination power with higher C-index values. All together, these results revealed that the nomogram constructed in this study can be used as a more powerful and simple-to-use tool to evaluate the OS and CSS prognosis for patients with VEO-CRC.

Several limitations of our study should be acknowledged. First, in this preliminary study, we selected a set of patients diagnosed with VEO-CRC from the SEER database and further randomly divided into the training cohort and validation cohort for model construction and internal validation. To ensure the general clinical applicability of the established nomogram, further investigations based on another independent prospective cohort are thus warranted. Second, other known prognostic factors, such as obesity, low-fiber intake, high consumption of red and processed meat, and little physical activity [5,37], were not contained in the SEER database. As a result, we could not assess the influence of these factors. Moreover, data concerning symptoms at diagnosis were also not available in the SEER database. Several vital prognostic factors, such as KRAS, BRAF, and microsatellite instability, were inaccessible in the SEER database. Therefore, in this study, these factors did not incorporate in the proposed nomogram. Third, detailed therapeutic information such as surgical procedures and chemotherapy regimens is lacking for the nomogram, which greatly affected survival outcomes. Recent studies are discussing the role of more intensive chemotherapy regimens such as triplets in young CRC patients [38,39]. Finally, due to the retrospective nature of this study, which may cause possible selection bias.

## CONCLUSION

We identified clinical variables associated with survival in VEO-CRC and then established a comprehensive and accurate nomogram to predict the 1-, 3-, and 5-year OS and CSS prognosis of patients with VEO-CRC. Compared with TNM stage and SEER stage, the nomogram exhibited a better predictive performance and can be used to assist clinicians to optimize individualized treatment plans for VEO-CRC patients.
